# Promoting Aging in Place in Social Housing

**DOI:** 10.1093/geroni/igaa057.2494

**Published:** 2020-12-16

**Authors:** Christine Sheppard, Tam Perry, Andrea Austen, Sander Hitzig

**Affiliations:** 1 Sunnybrook Research Institute, Toronto, Ontario, Canada; 2 Wayne State University, Detroit, Michigan, United States; 3 City of Toronto, Toronto, Ontario, Canada; 4 Sunnybrook Hospital, North York, Ontario, Canada

## Abstract

As cities around the globe plan for current and future older cohorts, there is a need to explore innovative housing models to help older adults age in place. This paper presents findings from an action-research academic/community partnership on a new service model at Toronto Community Housing, the second largest social housing landlord in North America and home to 27,000 older adults. As Toronto works to improve delivery of housing/support services, more knowledge was needed to understand the inadequate and inconsistent delivery of services to tenants. Interviews/focus groups with older tenants and service providers (N=116) identified challenges related to unit condition (e.g., pest control) and tenancy management (e.g., arrears), and that the fragmentation of housing and health services negatively impacts older tenants’ abilities to access supports and age in place. The presentation will conclude with discussion of planning and policy decision making approaches relevant to both Canadian and American contexts.

